# Genetic Variation, Heritability, and Diversity Analysis of Upland Rice (*Oryza sativa* L.) Genotypes Based on Quantitative Traits

**DOI:** 10.1155/2015/290861

**Published:** 2015-07-15

**Authors:** Mst. Tuhina-Khatun, Mohamed M. Hanafi, Mohd Rafii Yusop, M. Y. Wong, Faezah M. Salleh, Jannatul Ferdous

**Affiliations:** ^1^Laboratory of Food Crops, Institute of Tropical Agriculture, Universiti Putra Malaysia (UPM), 43400 Serdang, Selangor, Malaysia; ^2^Bangladesh Rice Research Institute, Gazipur 1701, Bangladesh; ^3^Laboratory of Plantation Crops, Institute of Tropical Agriculture, Universiti Putra Malaysia (UPM), 43400 Serdang, Selangor, Malaysia; ^4^Department of Land Management, Institute of Tropical Agriculture, Universiti Putra Malaysia (UPM), 43400 Serdang, Selangor, Malaysia; ^5^Department of Plant Protection, Faculty of Agriculture, Universiti Putra Malaysia, 43400 Serdang, Selangor, Malaysia; ^6^Department of Biotechnology and Medical Engineering, Universiti Teknologi Malaysia, 81310 Skudai, Johor, Malaysia

## Abstract

Upland rice is important for sustainable crop production to meet future food demands. The expansion in area of irrigated rice faces limitations due to water scarcity resulting from climate change. Therefore, this research aimed to identify potential genotypes and suitable traits of upland rice germplasm for breeding programmes. Forty-three genotypes were evaluated in a randomised complete block design with three replications. All genotypes exhibited a wide and significant variation for 22 traits. The highest phenotypic and genotypic coefficient of variation was recorded for the number of filled grains/panicle and yields/plant (g). The highest heritability was found for photosynthetic rate, transpiration rate, stomatal conductance, intercellular CO_2_, and number of filled grains/panicle and yields/plant (g). Cluster analysis based on 22 traits grouped the 43 rice genotypes into five clusters. Cluster II was the largest and consisted of 20 genotypes mostly originating from the Philippines. The first four principle components of 22 traits accounted for about 72% of the total variation and indicated a wide variation among the genotypes. The selected best trait of the number of filled grains/panicle and yields/plant (g), which showed high heritability and high genetic advance, could be used as a selection criterion for hybridisation programmes in the future.

## 1. Introduction

Upland rice has been gaining popularity, because current high-yielding varieties have led to an increase in genetic vulnerability, a scarcity of water for irrigation, and a breakdown of resistance genes against emerging races of pathogen due to intensive cultivation. In recent decades, the increases in world rice production that have resulted from successes in research and the transfer of modern technology have mainly concerned irrigated high-yielding varieties. Research into upland rice has been very limited and most of the research findings have not been published; therefore, these successes have had virtually no effect on upland rice production. Upland rice comprises 11% of the total global rice production and is cultivated on about 14 million hectares [1]. It is also important in cropping systems, because of the lack of irrigation facilities and lower cost of production [2].

In Brazil, the mean yield of upland rice is about 2 t/ha, compared to about 5 t/ha for lowland or irrigated rice [3]. In Nigeria, although the production of rice has increased due to a marked expansion in area, the productivity per unit area has remained at about 1.5 t/ha [4]. Generally, the grain yields of upland rice are low, from 0.5 to 1.5 t/ha in Asia, about 0.5 t/ha in Africa, and from 1.0 to 4.0 t/ha in Latin America [5]. The area planted with rice in Malaysia is estimated to be 672,000 ha and the mean national rice production is 3.66 metric tones/ha [6], of which upland rice is cultivated on about 165,888 ha, with a mean yield ranging from 0.46 to 1.10 t/ha [1].

To boost the yield potential of upland rice, it is necessary to identify cultivars with improved yield and other desirable agronomic characters, to overcome the global problem of hunger and starvation, especially in African countries [7]. Genetic variation is the basis of plant breeding and provides a great array of genotypes that can be selected to develop new varieties or breeding materials [8]. Variability in terms of genetic divergence for agronomic traits is the key component of breeding programmes for broadening the gene pool of rice and requires reliable estimates of heritability to plan an efficient breeding programme [9]. Knowledge concerning heritability helps plant breeders to predict the nature of the succeeding generation, to make an appropriate selection and to assess the magnitude of genetic improvement through selection [10]. This study was therefore conducted to select potential genotypes and to identify the most important characters for breeding programmes by exploiting the genetic variation, heritability, and diversity analysis of yield and related attributes of 43 upland rice genotypes.

## 2. Materials and Methods

### 2.1. Experimental Location, Experimental Design, and Planting Material

The experiment was conducted at the net house of the experimental field #10, at the Universiti Putra Malaysia (UPM) from August to December 2013. The experimental site is located between 3°02′N and 101°42′E at an altitude of 31 m above sea level. The study was laid out as a randomised complete block design (RCBD) with three replications. Metal tanks were used for planting the rice genotypes. The size of the tank was 1.85 m × 1.24 m × 0.45 m. The tank was filled with well-pulverised soil.

### 2.2. Rice Genotypes, Raising of Seedlings, Transplantation, and Management Practices

Forty-three upland rice genotypes (Table 1) obtained from the International Rice Research Institute (IRRI) and Bangladesh Rice Research Institute (BRRI) were used in this study. Thirty sun-dried seeds of each genotype were treated with 70% ethanol for 90 s to control seed-borne diseases and were placed on moist filter paper in Petri dishes for germination. After germination, ten-day-old seedlings were transplanted into the tank. Only one seedling per hill was transplanted and each replicate consisted of two hills for each genotype. The interplant and interrow distance was maintained at 20 cm. Management practices such as irrigation, fertilization, weeding, and spraying were performed by following the standard procedures of the IRRI [11].

### 2.3. Data Collection

The following parameters were considered for data collection: days to flowering (DF, days), days to maturity (DM, days), plant height (PH, cm), flag leaf length (FL, cm), leaf chlorophyll content at 67 and 97 days after transplanting (CC67 and CC97), photosynthetic rate (*P*_*N*_, *μ*mol/m/s), transpiration rate (*E*, mmol/m/s), stomatal conductance (*g*_*s*_, mmol/m/s), intercellular CO_2_ (*C*_*i*_, ppm), total number of tillers/plant (TT, no), number of effective tillers/plant (ET, no), panicle length (PL, cm), number of filled grains/panicle (FG, no), number of unfilled grains/panicle (UFG, no), grain length (GL, mm), grain breadth (GB, mm), grain length/breadth ratio (*L*/*B*), straw yield/plant (SY, g), harvest index (HI, %), 100-grain weight (100 GW, g), and yield/plant (*Y*, g). The data were recorded based on a standard evaluation system (SES) introduced by the International Rice Research Institute [12].

### 2.4. Statistical Analyses

The analysis of variance and comparison of means were performed by SAS software version 9.2. Diversity analysis was conducted by NTSYS-PC software (version 2.1) and Minitab software (version 15). The genetic parameters, including the genotypic and phenotypic variance, genotypic and phenotypic coefficient of variance, heritability (broad sense), and the expected genetic advance (GA), were calculated using the formula given by Burton and DeVane (1953) and Johnson et al. (1955) [13, 14].

## 3. Results

### 3.1. Determination of Genetic Variations

The results revealed a wide range of variability among 43 upland rice genotypes for 22 quantitative traits (Table 2). The phenotypic variance (*σ*2P) of all traits was higher than the genotypic variance (*σ*2G); similarly, the phenotypic coefficient of variation (PCV) was also higher than genotypic coefficient of variation (GCV). The highest PCV was recorded for the traits FG and *Y*, which were 72.62 and 84.36%, respectively. In contrast, the lowest PCV belonged to the characters CC67 (7.00%) and CC97 (8.25%). The GCV ranged from 4.62 (CC67) to 80.07% (*Y*). The next highest GCV contained the characters FG (69.73%), HI (61.60%), TT (47.17%), and SY (41.31%). Similarly, CC97 (5.78%), GL (7.87%), DM (8.14%), and DF (11.14%) were the traits observed for the next-lowest GCV. Most of the traits in the present study exhibited a high heritability. A heritability of 100% was recorded for the characters *P*_*N*_, *E*, *g*_*s*_, and *C*_*i*_. Furthermore, a higher heritability was also recorded for FG (92.21%), *Y* (90.09%), and GB (85.71%). In this study, genetic advance (GA) was also calculated and ranged from 6.28% for CC67 to 156.56% for *Y*.

### 3.2. Cluster Analysis

**Figure 1:**
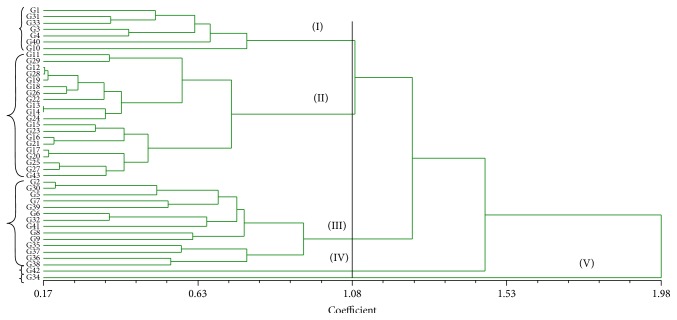
Cluster analysis of 43 upland rice genotypes based on morphological, physiological, and yield associated characters.

The Euclidean distance was calculated using standardised morphological data and a UPGMA dendrogram was constructed using these values for 43 upland rice genotypes. Five major groups were observed among 43 upland rice genotypes based on multivariate analysis at a 1.08 dissimilarity coefficient value (Figure 1). The value of 1.08 was set only for the convenience of explanation in this case. Cluster II contained the maximum number of genotypes (20), which consisted of 46.51% of all genotypes, mostly originating from the Philippines. The second highest was cluster III, which consisted of 14 members. Clusters I, IV, and V consisted of seven, one, and one genotypes, respectively.

### 3.3. Principal Component Analysis

**Figure 2:**
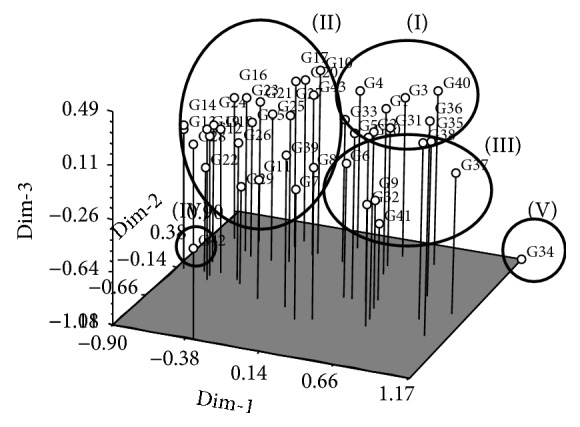
Three-dimensional graph of 43 upland rice genotypes based on morphological, physiological, and yield associated characters (principal component analysis).

The results of PCA partly confirmed the findings of cluster analysis: in PCA, the genotypes also clustered into five groups (Figure 2), with only few differences between groups I and III compared to cluster analysis. This demonstrates that the data obtained from this experiment were accurate, precise, and reliable. The PCA analysis showed that the first four principal components accounted for about 72.1% of the total variation and exhibited a very high correlation among them. The first, second, third, and fourth principle components explained about 35.7%, 53.1%, 64.5%, and 72.1% of the variation observed in the eigenvector analysis (Table 3). In the first PC: PH (0.29), FL (0.29), PL (0.29), FG (0.31), and HI (0.30) were the most important contributing traits; similarly, *P*_*N*_ (0.43), *E* (0.40), *g*_*s*_ (0.39), and GB (0.40) were the important parameters of the second PC.

## 4. Discussion

The genetic analysis of quantitative traits is a prerequisite for plant breeding programmes, which can lead to a systemic method of design and to the appropriate planning of plant breeding strategies. The current study suggests that the PCV was higher than the GCV for all traits. This was also the case for all the traits observed in another study [7], which reported that the environmental effect on any trait is indicated by the magnitude of the differences between the genotypic and phenotypic coefficients of variation; large differences reflect a large environmental effect, whereas small differences reveal a high genetic influence. In this study, the small differences between the PCV and GCV for most of the traits, such as DF, DM, *P*_*N*_, *E*, *g*_*s*_, *C*_*i*_, and GL, represented some degree of environmental influence on the phenotypic expression of these characters. It also suggests that selection based on these characters would be effective for future crossing programmes. The other traits, which showed a higher difference between PCV and GCV, indicated that the environmental effect on the expression of those traits is higher and that selection based on these characters is not effective for further yield improvement. The highest PCV was recorded for FG and *Y*. High GCV and PCV for FG and *Y* were also recorded by the following researchers [15–18]. The variables *P*_*N*_, *E*, *g*_*s*_, and *C*_*i*_ revealed 100% heritability, which indicated the presence of additive gene action. This also showed that selection based on these characters would be more effective and efficient than the use of other traits for segregating generations in future breeding programmes, which indicated an exploitable amount of variation. A study conducted by Jahn et al. [19] reported that the percentage variance for photosynthesis was highest among 20 diverse rice varieties based on 14 quantitative traits. However, they observed a low variance for transpiration and stomatal conductance. Genetic variation and diversity studies for the aforementioned traits are scarce for upland and lowland rice. However, many researchers have observed the *P*_*N*_, *E*, and *g*_*s*_ capacity of rice varieties under drought stress and control conditions and showed that all these parameters decrease under water stress in rice [20, 21]. The FG and *Y* showed a very high heritability coupled with the highest genetic advance, suggesting that these traits were mainly under genetic control and that they can be scored by their phenotypic performance. In a separate study of 19 upland rice accessions, Vange [4] estimated the heritability and genetic advance to be high, for the parameters days to 50% heading, days to maturity, flag leaf area, panicle weight, panicle length, no branches per panicle, no seeds/panicle, and seed weight/panicle at seed yield. High heritability (more than 60%) and high genetic advance (more than 20%) for leaf chlorophyll content, number of productive tillers per plant, panicle weight and number of grains per panicle, and 1,000-grain weight were recorded by Laxuman et al. [22].

The 43 upland rice genotypes clustered into five groups based on the dendrograms of cluster analysis and PCA. The groupings of hierarchical cluster analysis exhibited a similar dendrogram topology and cluster membership to that produced using PCA analysis, thereby confirming the accuracy of the constructed dendrogram. Likewise, Worede et al. [23] calculated a similar clustering pattern for hierarchical cluster analysis and principal component analysis for 24 rice genotypes. The multivariate analysis of the quantitative traits identified the most similar genotypes as 13 × 14 and 12 × 28, based on the dissimilarity coefficient, suggesting that hybridisation within these genotypes will not be effective, as their genetic make-up is almost similar. However, crossing between genotypes 13 × 34 and 14 × 34 will be effective, as these genotypes were identified as being most divergent. The Euclidian distance showed that the clusters were mostly formed based on the origin or geographical area of the genotypes. The genotypes from the same geographical origin mostly grouped together; however, the less frequent genotypes from different origins also grouped within the same cluster. Fifty-eight rice varieties grouped into four clusters based on 18 morphological characters in a study by Ahmadikhah et al. [24]. Rahman et al. [25] also divided 21 rice varieties into five clusters based on 14 physiological traits. Principal component analysis helps to understand how the genotypes of similar categories group together compared to dissimilar ones. The results of PCA can clarify or verify the cluster analysis; if the results of one analysis support those of another, this confirms that the data are more precise and accurate. The presence of strong differences among 43 upland rice genotypes in the present study was also further confirmed by PCA. The first four principal components accounted for 72% of the total variation, which indicated a very strong correlation among the characters being studied. The first PC, which solely contributed to 35.7% of the variation, was the most significant. Accordingly, in the first PC, the traits PH, FL, PL, FG, and HI were important in separating the genotypes due to their high loadings. Similarly, Worede et al. [23] explained 61.2% of the total variability using the first and second PCs. Approximately, 82.7% of the total variation among 32 upland rice varieties was also noted by Lasalita-Zapico et al. [26]. For the selection of parents, genetic diversity is one of the important decisive factors [15]. Our investigation highlighted that genotypes originating from the Philippines could be hybridised with genotypes from Malaysia, Thailand, Vietnam, India, or Bangladesh and vice versa, to create a broader genetic variation for yield and other favorable characters.

## 5. Conclusions

The present study showed the existence of a considerable level of diversity among the 43 upland rice genotypes. The highest heritability recorded for the parameters photosynthetic rate, transpiration rate, and number of filled grains/panicle and yields/plant demonstrates that these traits could be successfully transferred to offspring, if selection for these characters is performed in the hybridisation programme. The aforementioned traits of number of filled grains/panicle and yields/plant showed a very high genetic advance, which implies that these characters could be used to select upland accession genotypes for a notable improvement in cultivation in changing environments, particularly under conditions of water scarcity in the tropics. Furthermore, crossing the genotypes IR 5533-13-1-1 × Beu E-Soo and IR 5533-14-1-1 × Beu E-Soo could result in heterotic expression and a large variability in the segregating generation.

## Figures and Tables

**Table 1:** Name, origin, grain color, and status of sample of 43 upland rice genotypes (“x” means no information about this material).

Code number	Genotype name	Origin	Seed coat color	Status of sample
G1	Daeng Se Leuad	Thailand	Purple	x
G2	Khao Gam (NIAW)	Thailand	Purple	Landrace/traditional cultivar
G3	258	Liberia	Red	Breeding and inbred line
G4	Black Banni	India	Red	Landrace/traditional cultivar
G5	Blau Noc	Vietnam	Red	Landrace/traditional cultivar
G6	Ble La	Vietnam	Red	Landrace/traditional cultivar
G7	Ble Lia Su	Vietnam	Red	Landrace/traditional cultivar
G8	Chirikata 2	India	Red	x
G9	Choke Tang	Vietnam	Red	x
G10	Chokoto 14	Brazil	Red	x
G11	IPPA	Bhutan	Red	Landrace/traditional cultivar
G12	IR 3257-13-56	Philippines	Red	Breeding and inbred line
G13	IR 5533-13-1-1	Philippines	Red	Breeding and inbred line
G14	IR 5533-14-1-1	Philippines	Red	Breeding and inbred line
G15	IR 5533-15-1-1	Philippines	Red	Breeding and inbred line
G16	IR 5533-50-1-10	Philippines	Red	Breeding and inbred line
G17	IR 5533-55-1-11	Philippines	Red	Breeding and inbred line
G18	IR 5533-56-1-12	Philippines	Red	Breeding and inbred line
G19	IR 5533-PP 854-1	Philippines	Red	Breeding and inbred line
G20	IR 5533-PP 856-1	Philippines	Red	Breeding and inbred line
G21	IR 9559-3-1-1	Philippines	Red	Breeding and inbred line
G22	IR 9559-4-1-1	Philippines	Red	Breeding and inbred line
G23	IR 9559-5-3-2	Philippines	Red	Breeding and inbred line
G24	IR 9559-PP 871-1	Philippines	Red	Breeding and inbred line
G25	IR 9669-22-2-6	Philippines	Red	Breeding and inbred line
G26	IR 9669-23-12-7	Philippines	Red	Breeding and inbred line
G27	IR 9669-PP 823-1	Philippines	Red	Breeding and inbred line
G28	IR 9669-PP 830-1	Philippines	Red	Breeding and inbred line
G29	IR 9669-PP 836-1	Philippines	Red	Breeding and inbred line
G30	Ja Hau	Thailand	Red	Landrace/traditional cultivar
G31	Ja La Shau	Thailand	Red	Landrace/traditional cultivar
G32	Ja Loy	Thailand	Red	Landrace/traditional cultivar
G33	Ja No Naq	Thailand	Red	Landrace/traditional cultivar
G34	Beu E-Soo	Thailand	Purple	Landrace/traditional cultivar
G35	C	Ivory Coast	Red	Released/improved/advanced cultivar
G36	Padi Beleong	Malaysia	Red	x
G37	Padi Kalopak	Malaysia	Red	x
G38	F1 seed (BR 16 × Karingam)	Malaysia	White	F1 seed
G39	BR 24	Bangladesh	White	Improved cultivar
G40	BR26	Bangladesh	White	Improved cultivar
G41	BRRI dhan42	Bangladesh	White	Improved cultivar
G42	BRRI dhan43	Bangladesh	White	Improved cultivar
G43	BRRI dhan48	Bangladesh	White	Improved cultivar

**Table 2:** Estimation of genetic parameters of 22 agronomic, physiological, yield, and yield contributing traits.

Traits	Mean	MSG	MSE	*σ*2G	*σ*2P	PCV (%)	GCV (%)	*h* ^2^_*B*_ (%)	GA (%)
DF	85.66	296.95	23.77	91.07	114.84	12.51	11.14	79.3	20.44
DM	116.58	287.93	17.53	90.13	107.66	8.90	8.14	83.72	15.34
PH	103.88	1053.97	76.71	325.75	402.46	19.31	17.37	80.94	32.2
FL	30.67	169.4	25.73	47.89	73.62	27.98	22.56	65.05	37.49
TT	10.24	85.03	6.38	23.33	29.71	53.23	47.17	78.53	86.11
CC67	41.13	15.52	4.68	3.61	8.29	7	4.62	43.55	6.28
CC97	42.79	24.71	6.34	6.12	12.46	8.25	5.78	49.12	8.35
*P*_*N*_	9.14	30.38	0	10.13	10.13	34.82	34.82	100	71.73
*E*	3.03	1.74	0	0.58	0.58	25.13	25.13	100	51.78
*g*_*s*_	0.11	0.004	0	0.001	0.001	28.75	28.75	100	59.22
*C*_*i*_	227.98	5375	0.4	1791.5	1791.9	18.57	18.57	99.98	38.24
SY	16.09	151.72	19.19	44.18	63.37	49.48	41.31	69.72	71.06
HI	21.26	540.16	25.55	171.53	197.08	66.03	61.60	87.04	118.40
ET	6.83	29.98	3.48	7.76	11.24	49.09	40.79	69.04	69.81
PL	21.04	57.34	12.45	14.96	27.41	24.88	18.38	54.58	27.98
FG	33.2	1653.29	45.3	535.99	581.29	72.62	69.73	92.21	137.94
UFG	35.03	693.11	92.54	200.19	292.73	48.84	40.39	68.39	68.81
GL	6.35	0.826	0.067	0.25	0.317	8.87	7.87	78.86	14.4
GB	2.39	0.394	0.02	0.12	0.14	15.66	14.49	85.71	27.64
*L*/*B*	2.7	0.429	0.034	0.13	0.164	15	13.35	79.27	24.49
100 GW	1.99	0.366	0.03	0.11	0.14	18.8	16.67	78.57	30.43
*Y*	4.44	39.33	1.39	12.64	14.03	84.36	80.07	90.09	156.57

Note: MSG, mean square of accessions; MSE, mean square of error; *σ*2G, genotypic variance; *σ*2P, phenotypic variance; PCV, phenotypic coefficient of variance; GCV, genotypic coefficient of variance; *h* ^2^_*B*_, heritability in broad sense; GA, genetic advance.

**Table 3:** Eigenvectors and eigenvalues of the first four principle components of 22 traits.

Variable	PC1	PC2	PC3	PC4
Eigenvalue	7.86	3.81	2.50	1.66
Variation (%)	35.7	17.4	11.4	7.6
Cumulative (%)	35.7	53.1	64.5	72.1

DF	−0.289	0.022	−0.033	−0.047
DM	−0.314	0.044	0.006	0.095
CC67	0.201	0.143	−0.051	0.076
CC97	0.237	0.224	−0.072	0.169
*P*_*N*_	−0.07	0.433	0.012	0.193
*E*	−0.128	0.398	−0.027	−0.127
*g*_*s*_	−0.152	0.393	−0.059	−0.187
*C*_*i*_	−0.077	−0.036	−0.125	−0.493
PH	0.293	0.029	−0.13	−0.106
FL	0.289	0.074	−0.065	−0.064
TT	−0.27	−0.021	−0.361	−0.001
ET	−0.145	−0.109	−0.517	−0.02
PL	0.291	0.078	−0.002	0.042
FG	0.313	−0.087	−0.037	−0.109
UFG	0.199	−0.08	0.076	0.011
GL	0.073	0.178	−0.194	0.506
GB	0.08	0.399	0.012	−0.171
*L*/*B*	−0.019	−0.322	−0.131	0.45
100 GW	0.152	0.275	−0.256	0.198
*Y*	0.217	−0.093	−0.404	−0.224
SY	−0.115	−0.008	−0.494	−0.009
HI	0.298	−0.092	−0.134	−0.13

Note: PC1, first principle component; PC2, second principle component; PC3, third principle component; and PC4, four principle component.
